# Mirogabalin Decreases Pain-like Behaviors by Inhibiting the Microglial/Macrophage Activation, p38MAPK Signaling, and Pronociceptive CCL2 and CCL5 Release in a Mouse Model of Neuropathic Pain

**DOI:** 10.3390/ph16071023

**Published:** 2023-07-19

**Authors:** Renata Zajączkowska, Katarzyna Pawlik, Katarzyna Ciapała, Anna Piotrowska, Agata Ciechanowska, Ewelina Rojewska, Magdalena Kocot-Kępska, Wioletta Makuch, Jerzy Wordliczek, Joanna Mika

**Affiliations:** 1 Department of Interdisciplinary Intensive Care, Jagiellonian University Medical College, 30-688 Krakow, Poland; j.wordliczek@uj.edu.pl; 2 Department of Pain Pharmacology, Maj Institute of Pharmacology Polish Academy of Sciences, 31-343 Krakow, Poland; pawlik@if-pan.krakow.pl (K.P.); kciapala@if-pan.krakow.pl (K.C.); anna.piotrowskamurzyn@gmail.com (A.P.); ciechan@if-pan.krakow.pl (A.C.); rojewska@if-pan.krakow.pl (E.R.); makuch@if-pan.krakow.pl (W.M.); 3 Department of Pain Research and Treatment, Jagiellonian University Medical College, 31-501 Krakow, Poland; magdalena.kocot-kepska@uj.edu.pl

**Keywords:** mirogabalin, pregabalin, microglia, neuropathic pain, p38MAPK, CCL2, CCL5

## Abstract

Neuropathic pain is a chronic condition that significantly reduces the quality of life of many patients as a result of ineffective pain relief therapy. For that reason, looking for new analgesics remains an important issue. Mirogabalin is a new gabapentinoid that is a specific ligand for the α2σ-1 and α2σ-2 subunits of voltage-gated calcium channels. In the present study, we compared the analgesic effect of pregabalin and mirogabalin in a neuropathic pain chronic constriction injury (CCI) of the sciatic nerve in a mouse model. The main purpose of our study was to determine the effectiveness of mirogabalin administered both once and repeatedly and to explain how the drug influences highly activated cells at the spinal cord level in neuropathy. We also sought to understand whether mirogabalin modulates the selected intracellular pathways (p38MAPK, ERK, JNK) and chemokines (CCL2, CCL5) important for nociceptive transmission, which is crucial information from a clinical perspective. First, our study provides evidence that a single mirogabalin administration diminishes tactile hypersensitivity more effectively than pregabalin. Second, research shows that several indirect mechanisms may be responsible for the beneficial analgesic effect of mirogabalin. This study reports that repeated intraperitoneally (i.p.) mirogabalin administration strongly prevents spinal microglia/macrophage activation evoked by nerve injury, slightly suppresses astroglia and neutrophil infiltration, and reduces the p38MAPK levels associated with neuropathic pain, as measured on Day 7. Moreover, mirogabalin strongly diminished the levels of the pronociceptive chemokines CCL2 and CCL5. Our results indicate that mirogabalin may represent a new strategy for the effective pharmacotherapy of neuropathic pain.

## 1. Introduction

Neuropathic pain is a difficult clinical problem. According to the International Association for the Study of Pain definition, it is a result of lesions or diseases of the somatosensory nervous system [1]. It is estimated that 7–10% of the general population suffers from this type of pain [2], which has a negative impact on patients’ physical and psychological health and wellbeing and lowers their quality of life [3]. One of the reasons for the negative impact of neuropathic pain on the quality of life of hundreds of millions of people worldwide is the unsatisfactory efficacy of currently available treatment methods. Studies show that only 30–50% of patients suffering from neuropathic pain obtain pain relief, and that treatment of the remaining patients, despite the use of various methods and techniques, is ineffective [4]. Therefore, new studies are being conducted to find drugs and methods of treatment that can be used clinically to improve this unfavorable situation. One of the drugs recently introduced into clinical practice for the treatment of neuropathic pain is mirogabalin. Mirogabalin is a new gabapentinoid that is a specific ligand for the α2σ-1 and α2σ-2 subunits of voltage-gated calcium channels (VGCCs). Compared to the older members of the gabapentinoid family, gabapentin and pregabalin, mirogabalin binds more strongly and with higher selectivity to the α2σ-1 and α2σ-2 subunits of voltage-gated calcium channels and exhibits a slower dissociation rate from α2σ-1 than from the α2σ-2 subunits of VGCCs [5]. The clinical result of these differences is greater analgesic efficacy and a lower rate of adverse effects of mirogabalin when compared to gabapentin and pregabalin [6].

Many papers suggest an essential role of glia and immune cell activation in the development of neuropathic pain [7]. There is a quickly growing body of evidence demonstrating that signaling from microglia [7,8,9,10,11], astroglia [7,12], and neutrophils [12] plays a crucial role in the pathogenesis of neuropathy. The abovementioned cells are known to be rapidly activated and may release pronociceptive molecules, including chemokines. Moreover, the results of several studies suggest that the spinal activation of Mitogen Activated Protein Kinases (MAPKs), namely p38, extracellular signal-regulated kinase (ERK), and c-Jun N-terminal kinase (JNK) plays a role in the early phase of neuropathic pain development [7,13,14,15] and contributes to the downstream activation of many nociceptive factors, including chemokines [7,16].

For that reason, we first compared the effect of a single intraperitoneal (i.p.) injection of pregabalin and mirogabalin on fully established tactile and thermal hypersensitivity in mice following chronic constriction injury (CCI) of the sciatic nerve (Bennett model). Second, we studied how preemptive and repeated (for 7 days) i.p. administration of mirogabalin influences hypersensitivity on Days 2, 5, and 7 in the neuropathic pain model. Our main goal was to assess whether and how repeated mirogabalin administration for 7 days influences the activation of microglia, astroglia, and neutrophils at the spinal cord level, and, in parallel, the selected intracellular factors (p38, ERK, JNK) and strongly pronociceptive chemokines.

## 2. Results

### 2.1. Effects of a Single i.p. Mirogabalin and Pregabalin Administration on Pain-Related Behavior Measured 11 Days after CCI in Mice

**Figure 1:**
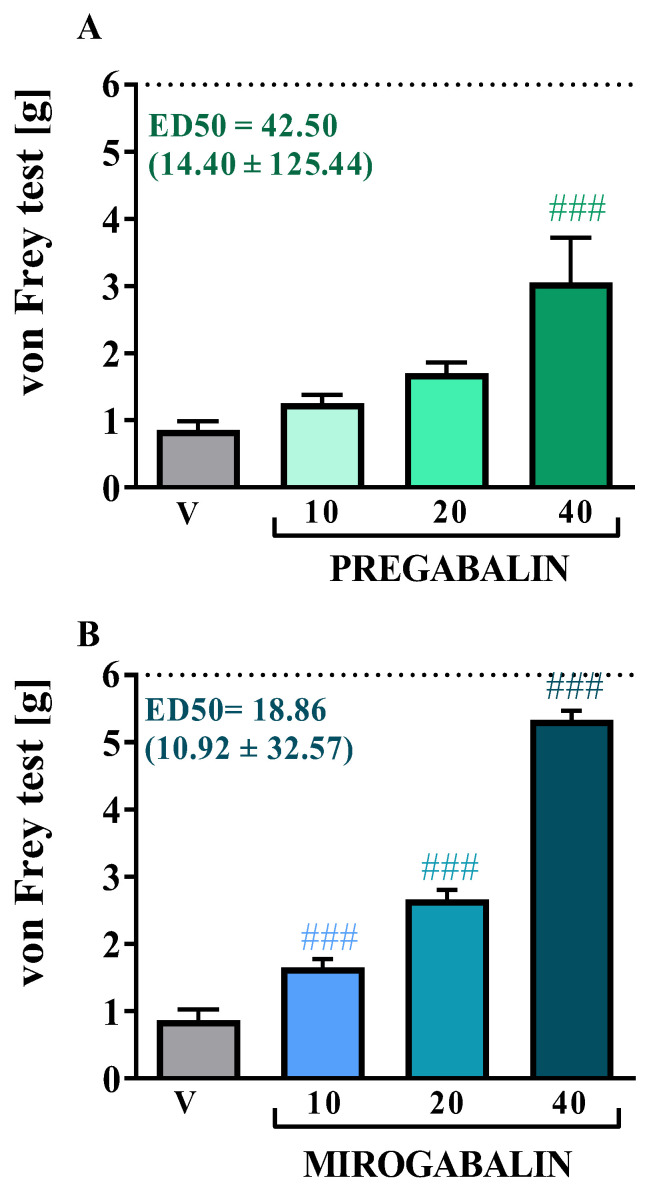
Effect of a single intraperitoneal (i.p.) administration of pregabalin (10, 20, 40 mg/kg) and mirogabalin (10, 20, 40 mg/kg) on pain-related behavior in mice on Day 11 post-CCI (von Frey test (**A**,**B**). The data are presented as the mean ± SEM (6–7 mice per group). The horizontal dotted line represents the cutoff value. Intergroup differences were analyzed using ANOVA with Bonferroni’s multiple comparisons post hoc test. ### *p* < 0.001 indicates a significant difference between V-treated and pregabalin-treated CCI-exposed animals and V-treated and mirogabalin-treated CCI-exposed animals. ED50 values were also calculated for both pregabalin and mirogabalin in the von Frey tests. **V, vehicle**.

A single i.p. administration of pregabalin at concentrations of 10, 20, and 40 mg/kg was performed 11 days after surgery, when hypersensitivity was fully developed in CCI-exposed mice. The influence of pregabalin on tactile hypersensitivity was measured by the von Frey (Figure 1A) test at 1 h after administration. The highest dose of pregabalin (40 mg/kg) showed the strongest analgesic effect in the von Frey test [F_(3, 24)_ = 7.329, *p* = 0.0012]. A single i.p. injection of pregabalin at lower doses (10 mg/kg and 20 mg/kg) did not provide any relief of pain-related behavior (Figure 1A).

A single i.p. administration of mirogabalin at doses of 10, 20, and 40 mg/kg was administered 11 days after CCI. The effect of mirogabalin administration on tactile hypersensitivity was measured using the von Frey (Figure 1B) test 1 h after drug administration. The greatest analgesic effect was demonstrated after administration at the highest dose of 40 mg/kg, significantly attenuating tactile hypersensitivity [F_(3, 21)_ = 190.1, *p* < 0.0001] (Figure 1B). Two lower doses also exhibit analgesic effect, but they had a less potent effect than for dose 40 mg/kg.

Importantly, the necessary dose of substance to produce a 50% response (ED50%) in the von Frey test was lower for mirogabalin when compared to pregabalin.

### 2.2. Effects of Repeated i.p. Mirogabalin Administration on Pain-Related Behavior Measured 2, 5 and 7 Days after CCI in Mice

**Figure 2:**
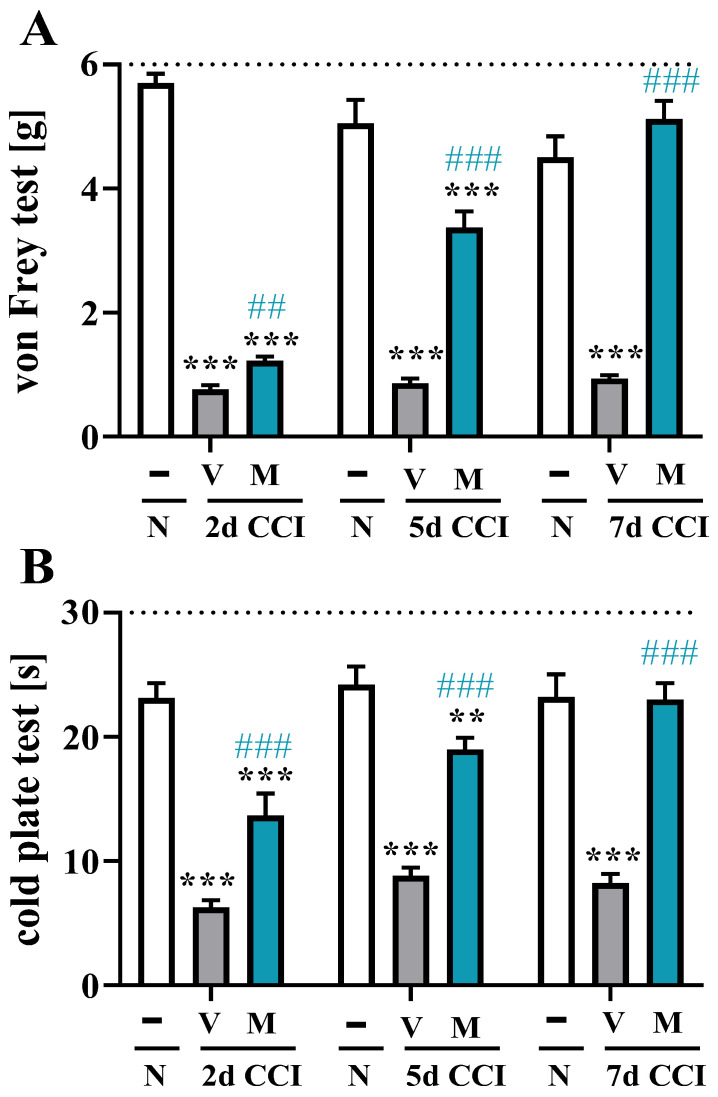
Effect of repeated intraperitoneal (i.p.) administration of mirogabalin (M; 20 mg/kg) 16 h and 1 h before CCI and then twice daily for 7 days on tactile (von Frey—(**A**)) and thermal hypersensitivity (cold plate—(**B**)) in CCI-exposed mice as measured on Days 2, 5, and 7 after chronic constriction injury. The horizontal dotted line represents the cutoff value. The data are presented as the mean ± SEM (8–10 mice per group). Intergroup differences were analyzed using ANOVA with Bonferroni’s multiple comparisons post hoc test. ** *p* < 0.01, *** *p* < 0.001 indicate differences between the naïve and V- or M-treated CCI-exposed mice; ## *p* < 0.01, ### *p* < 0.001 indicate differences between V- and M-treated CCI-exposed mice. **CCI, chronic constriction injury; N, naïve; V, vehicle; M, mirogabalin**.

All CCI-exposed mice developed tactile (*p* < 0.0001; Figure 2A) and thermal (*p* < 0.0001; Figure 2B) hypersensitivity when compared to the control (naïve) group. Mirogabalin (20 mg/kg, i.p.) was injected 16 h and 1 h before CCI and then twice per day for 7 days after injury. The influence of mirogabalin administration on the development of hypersensitivity to tactile and thermal stimuli was measured 2, 5, and 7 days following CCI using von Frey (Figure 2A) and cold plate (Figure 2B) tests, respectively, 1 h after drug administration. Repeated i.p. administration of mirogabalin evoked analgesic effects in CCI-exposed mice at all tested time points in both tests. However, the greatest analgesic effect after mirogabalin injection was observed 7 days post-CCI in comparison with that of vehicle-treated animals in the von Frey [F_(2, 25)_ = 78.46, *p* < 0.0001] and cold plate [F_(2, 25)_ = 40.05, *p* < 0.0001] tests (Figure 2A,B).

### 2.3. Effect of the Repeated Administration of Mirogabalin on IBA-1, GFAP and MPO Protein Levels in the Spinal Cord Measured on Day 7 after CCI in Mice

**Figure 3:**
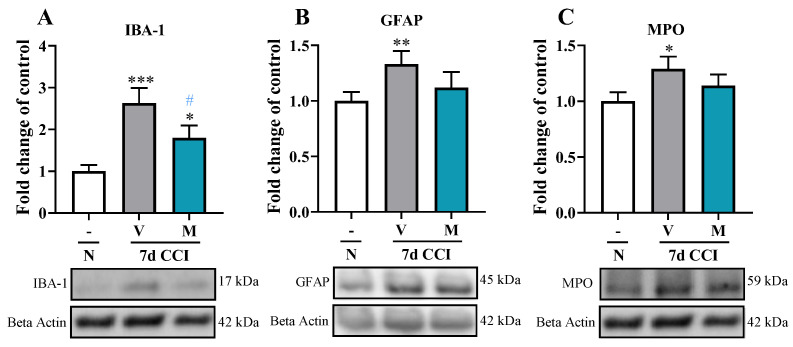
Effect of a repeated intraperitoneal (i.p.) administration of mirogabalin (M; 20 mg/kg) on IBA-1 (**A**), GFAP (**B**) and MPO (**C**) protein levels in the spinal cord on Day 7 post-CCI in mice. The Western blot data are presented as the fold change ± SEM of 7–8 samples per group. Intergroup differences were analyzed based on one-way ANOVA with Bonferroni’s multiple comparisons post hoc test. * *p* < 0.05; ** *p* < 0.01; *** *p* < 0.001 indicate a significant difference compared with the naïve group. # *p* < 0.05; indicates differences compared with V-treated CCI-exposed mice. **CCI, chronic constriction injury; N, naïve; V, vehicle; M, mirogabalin**.

Chronic constriction injury primarily led to a strong increase in the protein level of IBA-1 (Figure 3A), and slight increase in GFAP (Figure 3B) and MPO (Figure 3C) was also observed. Moreover, mirogabalin treatment significantly inhibits the CCI-evoked upregulation of the level of microglia/macrophage marker [F_(2, 17)_ = 8.73, *p* = 0.0025] (Figure 3A) with no changes observed in the case of astroglia (Figure 3B) and neutrophil (Figure 3C) markers. However, despite the lack of changes between the vehicle- and mirogabalin-treated groups, the protein level of GFAP and MPO remained at the similar level as that in naïve animals.

### 2.4. Effects of Repeated i.p. Administration of Mirogabalin on pp38/p38, pERK/ERK, and pJNK/JNK Protein Levels in the Spinal Cord Measured on Day 7 after CCI in Mice

**Figure 4:**
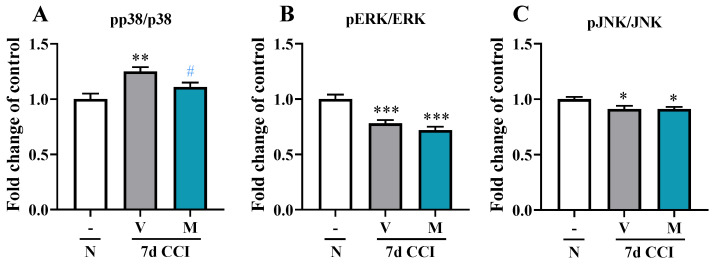
Changes in the protein levels of pp38/p38 (**A**), pERK/ERK (**B**), and pJNK/JNK (**C**) in the spinal cord on Day 7 post CCI after repeated vehicle or mirogabalin (M; 20 mg/kg i.p.) administration, measured using Luminex Assays. Data are presented as the mean ± SEM (n = 7–9 samples per group). Intergroup differences were analyzed based on one-way ANOVA with Bonferroni’s multiple comparisons post hoc test. * *p* < 0.05; ** *p* < 0.01; *** *p* < 0.001 indicate a significant difference compared with the naïve group. # *p* < 0.05 indicates differences compared with V-treated CCI-exposed mice. **CCI, chronic constriction injury; N, naïve; V, vehicle; M, mirogabalin**.

Sciatic nerve injury led to an increase of protein levels of pp38/p38 (Figure 4A). Interestingly, mirogabalin significantly inhibited the increase in pp38/p38 levels [F_(2, 21)_ = 8.702, *p* = 0.0018]. However, the levels of pERK/ERK and pJNK/JNK protein were significantly decreased in vehicle-treated CCI model mice when compared with naïve mice. In contrast, the levels remained unchanged in mirogabalin-treated CCI-exposed mice (Figure 4B,C).

### 2.5. Effects of Repeated i.p. Administration of Mirogabalin on CCL2 and CCL5 Measured on Day 7 after CCI in the Spinal Cord in Mice

**Figure 5:**
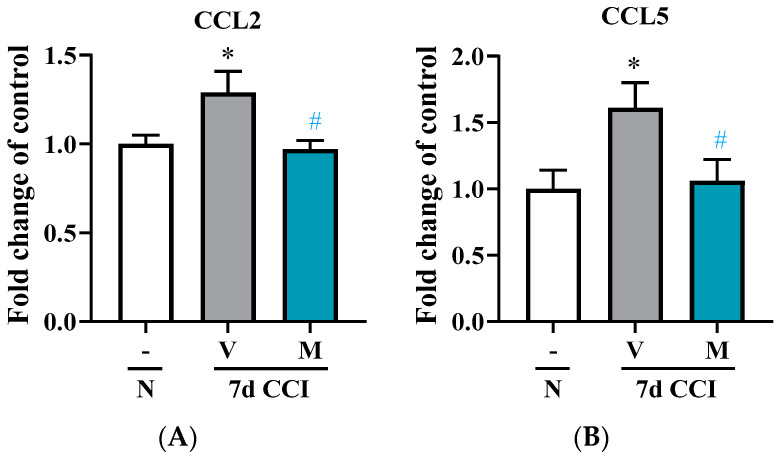
Changes in the protein levels of CCL2 (**A**) and CCL5 (**B**) in the spinal cord on Day 7 post CCI after repeated vehicle or mirogabalin (M; 20 mg/kg i.p.) administration, measured using Luminex Assays. Data are presented as the mean ± SEM (n = 5–8 samples per group). Intergroup differences were analyzed based on one-way ANOVA with Bonferroni’s multiple comparisons post hoc test. * *p* < 0.05 indicates differences between naïve and V-treated CCI-exposed rats; # *p* < 0.05 indicates differences between V-treated CCI-exposed mice. **CCI, chronic constriction injury; N, naïve; V, vehicle; M, mirogabalin**.

The CCL2 protein level was strongly increased in the spinal cord 7 days after CCI compared with naïve mice. Mirogabalin significantly reversed this change [F_(2, 17)_ = 4.454, *p* = 0.0278, Figure 5A]. Moreover, the elevated CCL5 protein level evoked by CCI was also decreased by mirogabalin administration [F_(2, 18)_ = 4.273, *p* = 0.0303, Figure 5B].

## 3. Discussion

In the present study, we demonstrated that single and repeated i.p. administration of mirogabalin reduces CCI-evoked hypersensitivity. Importantly, our research shows that mirogabalin relieves tactile hypersensitivity to a greater extent than pregabalin, a gabapentinoid used as the first-line drug in neuropathic pain therapy. Importantly, similar to our results, others also proved that mirogabalin had robust analgesic effects after it was orally administered to CCI-exposed rats [17]. Additionally, those authors showed that mirogabalin also diminished anxiety-related behavior [17]. The analgesic potential of mirogabalin was recently confirmed in experimental models of fibromyalgia [18], posttraumatic trigeminal neuropathy [19], and inflammatory pain evoked by formalin [20]. Moreover, mirogabalin appears to be effective after spinal cord injury in rats [5] and humans [21]. In January 2019, mirogabalin was approved in Japan for the treatment of peripheral neuropathic pain following promising clinical trials in patients with postherpetic neuralgia and painful diabetic polyneuropathy [22]. Importantly, in 2021, Japanese researchers published a study that showed that switching from pregabalin to mirogabalin is well tolerated and effective in pain management for peripheral neuropathic pain using stepwise titration [23]. Importantly, the analgesic properties of mirogabalin have been well confirmed in several experimental studies of pain of various etiologies, and more recently in clinical trials; however, the mechanism of its action has not been fully explained. In this study, we performed experiments on 4–5 weeks old mice, but it would be also interesting to clarify whether mirogabalin is also effective in older mice, especially since literature data indicate that neuropathic pain affects more often elderly patients [24] who are at high risk of polypharmacy [25]. In our study, we decided to focus on the molecular mechanism of action of mirogabalin.

The role of spinal glia in neuropathy was first suggested when enhanced levels of glial fibrillary acidic protein (GFAP; an astrocyte marker) and integrin αM (OX-42; a microglia marker) were found in neuropathic pain models [26,27]. Currently, it is well established that the activation of glial (astroglia, microglia) and immune (macrophages, neutrophils) cells is one of the reasons for the development and maintenance of neuropathic pain [7,28]. In CCI-exposed mice, we showed strong spinal activation of microglia [29,30], astroglia [29,30], and neutrophils [31] on day 7. Therefore, we chose that time point to study whether mirogabalin influences the activation of those cells. Our Western blot analysis provides the first evidence that, in CCI-induced neuropathy, repeated mirogabalin administration reduces the spinal level of IBA-1, a microglia/macrophage marker. Similarly, gabapentin in streptozotocin-induced diabetic neuropathic pain decreased hypersensitivity and simultaneously diminished spinal microglial activation; as in our study, it did not influence astroglia marker [32]. Moreover, gabapentin administered intrathecally decreased spinal microglia in rats [33,34] but had no impact on astroglia [34] in a complete Freund adjuvant monoarthritic model [34]. It remains to be clarified whether mirogabalin directly affects microglia/macrophages or whether these observed changes in the activation of these cells result from its effect on neuronal cells. Given the rapid analgesic effect of mirogabalin after its single dose, we believe that this drug produces analgesia directly through receptors localized on neuronal cells, which has already been suggested by some authors [6,35,36]. For example, in an in vitro study, mirogabalin inhibited N-type calcium channel currents in rat neurons [36]. Notably, mirogabalin inhibited the calcium channel currents of neurons at 50 μM and pregabalin at 200 μM [36]; this correlates well with the analgesic results obtained in the von Frey test, since we observed a lower ED50 for mirogabalin than for pregabalin. Therefore, we believe that mirogabalin may be more effective than pregabalin in patients, and we hope that, similar to pregabalin, mirogabalin reduces neuronal activation and damage [37]. Moreover, mirogabalin has better selectivity and a slower dissociation rate for α2δ-1 and α2δ-2 subunits of VGCCs when compared to gabapentin and pregabalin, which may result in its good analgesia, better safety profile, and relatively lower incidence of adverse effects when compared to gabapentin and pregabalin [38].

To explain the molecular basis of the observed analgesia, we studied the impact of mirogabalin on the mitogen-activated protein kinase family (MAPK family), which consists of three primary members: p38, ERK, and JNK. Importantly, for the first time, we showed that mirogabalin is able to diminish pp38/p38 levels, the kinase that is known to be essential for pain development after nerve injury [39,40,41]. In contrast, mirogabalin did not influence the levels of pERK/ERK and pJNK/JNK after nerve injury on day 7. This is in line with published data showing that, in mice, pERK/ERK levels increase after sciatic nerve injury in the early phase (day 1 to 3) [42]. pJNK/JNK is present primarily in astroglia [43] in the later phase (14 days) after nerve injury [44]. The importance of p38 for nociception processes has already been proven by numerous pharmacological experiments, in which the administration of p38 inhibitors (e.g., SB203580, CNI-1493, FR167653) prevented and/or reversed neuropathic pain symptoms [11,13,30,39,45,46,47,48,49]. Moreover, it was proven that phosphorylated p38 is mainly enhanced in spinal microglia after nerve injury but not in neurons and astrocytes [13,39,41,49,50]. Our study shows, for the first time, that p38 is involved in mirogabalin analgesia; however, this effect is probably indirect, and this result requires further study. Importantly, it is well established that p38 in microglial cells [7] is crucial for the intracellular signaling that leads to the production of many nociceptive factors responsible for neuropathic pain development [7]. In our study, we have shown that the levels of two highly pronociceptive chemokines that change in neuropathy, CCL2 [51] and CCL5 [52], did not increase in animals repeatedly receiving mirogabalin. In summary, our and others’ results provide evidence that mirogabalin has a unique and broad spectrum of action.

## 4. Materials and Methods

### 4.1. Animals

Adult male Albino-Swiss CD-1 mice (age 4–5 weeks, weighing 20–25 g), purchased from Charles River Laboratories (Hamburg, Germany), were placed in sawdust-lined cages, ten per cage, under controlled conditions (temperature 21 ± 2 °C; 12 h light/dark cycle; light on at 6 a.m.) and provided food and water as needed. All experiments were performed in accordance with the principles set by the International Association for the Study of Pain [53] and the NIH Guidelines for the Care and Use of Laboratory Animals. The study was approved by the Local Ethics Committee Branch II, National Ethics Committee for Experiments on Animals, Maj Institute of Pharmacology, Polish Academy of Sciences (approval no. 236/2020 Krakow, Poland). Precautions were taken to minimize animal suffering as well as the number of animals used (3R policy).

### 4.2. Sciatic Nerve Surgery

Chronic constriction injury to the sciatic nerve was performed under isoflurane anesthesia according to the procedure described by Bennett and Xie [54] and modified for mice by Mika et al. [29]. An incision was made below the right hip bone to expose the sciatic nerve. Once the sciatic nerve was exposed, three ligatures (3/0 silk) were made around it distal to the sciatic notch with 1-mm spacing until a brief twitch in the respective hind limb was noted. Afterward, long-lasting thermal and tactile hypersensitivity was observed. Control animals were not subjected to the procedure.

### 4.3. Drug Administration

The following drugs were used in the present study: mirogabalin (M; 10, 20, 40 mg/kg, MedChemExpress, Monmouth Junction, USA), and pregabalin (10, 20, 40 mg/kg, Pfizer Inc., New York, NY, USA). The scheme of the experiments and dosages used in our current study were based on our previously published papers [55,56,57]. The drugs were dissolved in aqua pro-injection and administered intraperitoneally (i.p.). The control group received vehicle (aqua pro-injection) according to the same protocol. Mirogabalin was administered in two experimental schedules: (1) a single injection on day 11 post CCI (10, 20, 40 mg/kg) and (2) repeated administration starting from 16 h and 1 h before CCI and then twice a day for 7 days (20 mg/kg). In the case of pregabalin, only a single injection of substance was performed on Day 11 postsurgery (10, 20, 40 mg/kg). No adverse side effects of mirogabalin and pregabalin treatment were noted during the experiments.

### 4.4. Behavioral Tests

Behavioral experiments were conducted between 8 a.m. and 12 p.m. The behavioral tests (von Frey test) were performed 1 h after mirogabalin and pregabalin treatment in the case of single injections. In the case of repeated administration of mirogabalin, behavioral tests (von Frey and cold plate tests) were also performed 1 h after drug injection on days 2, 5, and 7 post-CCI.

#### 4.4.1. Von Frey Test

Tactile hypersensitivity to non-noxious stimuli was measured using the von Frey test as previously described [29]. A set of calibrated nylon monofilaments (0.6–6 g; Stoelting, Wood Dale, IL, USA) were incrementally applied to the midplantar surface of mice. The ipsilateral hind paw of CCI-exposed mice and both paws in the case of naïve animals were measured until a response consistent with pain behavior was noted. The latter comprised rapid paw withdrawal, shaking, and licking. In the von Frey test, the findings are expressed as pressure [g] applied with the filament; the cutoff value was 6 g.

#### 4.4.2. Cold Plate Test

The response to noxious stimuli in the naïve and CCI-exposed animals was evaluated using a Cold/Hot Plate Analgesia Meter (No. 05044, Columbus Instruments, Columbus, OH, USA) as previously described [29]. The temperature of the plate was kept at 2 °C, and the cutoff value was 30 s. The mice were placed on the cold plate and the time until the injured paw was lifted was recorded. In CCI-exposed mice, the injured (right) paw always responded first. Therefore, the effect of the treatment is presented as the reaction of the ipsilateral hind paw.

### 4.5. Western Blot

Tissues from ipsilateral spinal cord segments (L4–L6) were collected from CCI-exposed and naïve mice after decapitation on the day 7 post-CCI, 6 h after mirogabalin administration. Next, samples were placed into RIPA buffer supplemented with a protease inhibitor cocktail (Sigma–Aldrich, St. Louis, MO, USA) and homogenized and fractioned via centrifugation as previously described [58,59,60]. First, the homogenate was centrifuged (2750 rpm, 5 min, 4 °C) to obtain the nuclear fraction (pellet). Second, the obtained supernatant was recentrifuged (8900 rpm, 5 min, 4 °C) to separate the mitochondrial fraction (pellet). Next, the supernatant was centrifuged once again using an ultracentrifuge (28.700 rpm, 60 min, 4 °C), which resulted in the separation of the cytosol (supernatant) and membrane (pellet). Cytosolic fractions were used for analyses. The total protein concentration was assessed using the bicinchoninic acid method. The obtained samples (10 µg of protein) were heated in a mix of loading buffer (4× Laemmli Buffer, Bio-Rad, Warsaw, Poland) containing 2-mercaptoethanol (Bio-Rad) for 8 min at 98 °C. Electrophoresis was performed using 4–15% Criterion™ TGX™ precast polyacrylamide gels (Bio-Rad). Next, the proteins were transferred (semidry transfer 30 min, 25 V) to Immune-Blot PVDF membranes (Bio-Rad) and then blocked for 1 h at RT using 5% bovine serum albumin (Sigma–Aldrich) in Tris-buffered saline containing 0.1% Tween-20 (TBST). After transfer, the membranes were washed with TBST and incubated overnight (4 °C) with the following primary antibodies: rabbit anti-IBA-1 (1:500; Novus), anti-GFAP (1:10,000; Novus), anti-MPO (1:1000; Abcam, Cambridge, UK), and mouse anti-beta actin (1:1000, Merck, Darmstadt, Germany). Afterward, the membranes were washed with TBST and incubated for 1 h at room temperature with HRP-conjugated anti-rabbit or anti-mouse secondary antibodies (1:5000, Vector Laboratories, Burlingame, CA, USA) diluted in buffer from a SignalBoost™ Immunoreaction Enhancer Kit (Merck). Proteins were detected using Clarity™ Western ECL Substrate (Bio-Rad) and visualized using a Fujifilm LAS-4000 FluorImager system. Fujifilm MULTI GAUGE software V3.0 (Tokio, Japan) software was used to estimate the intensities of the immunoreactive bands.

### 4.6. MILLIPLEX Multiplex Assays Using Luminex^®®^ to Analyze Protein Levels

Tissue samples from the lumbar (L4–L6) spinal cord were collected and prepared for analysis in the same manner as described in the Western blot (Analysis of Protein Levels). The protein concentrations of CCL2 and CCL5 were used in a MILLIPLEX^®®^ MAP Mouse Cytokine Chemokine Magnetic Bead Panel Immunology Multiplex Assay (Merck Millipore, Burlington, MA, USA) according to the manufacturer’s instructions. Similarly, p38, pp38, ERK, pERK, JNK, and pJNK were evaluated using the MILLIPLEX^®®^ MAP Multi-Pathway 9-plex Magnetic Bead Kit and MILLIPLEX^®®^ 9-plex Multi-Pathway Total Magnetic Bead Kit (Merck Millipore, Burlington, MA, USA) in accordance with the provided protocols.

### 4.7. Statistical Analysis

The behavioral test analyses are presented in grams and seconds as the mean ± standard error of the mean (SEM). One-way analysis of variance (ANOVA) was used to evaluate the experimental results. Differences between groups were analyzed with Bonferroni’s post hoc test. The data obtained from biochemical analyses are presented as the fold change compared with naïve mice on the ipsilateral side of the lumbar spinal cord. The biochemical analyses are presented as the mean ± SEM, which represents normalized averages. The intergroup differences were analyzed using ANOVA with Bonferroni’s multiple comparisons post hoc test. Moreover, computerized Litchfield and Wilcoxon methods were used to determine the antinociceptive dose necessary to produce a 50% response (ED50) and the 95% confidence limits on the quantal data. All of the statistical analyses were performed using Prism (ver. 8.1.1 (330), GraphPad Software, Inc., San Diego, CA, USA).

## 5. Conclusions

Our study showed that mirogabalin has a strong analgesic effect on neuropathic pain that is even greater than that of the commonly used drug pregabalin. Our study provides the first evidence that repeated i.p. mirogabalin administration prevents the microglia/macrophages activation evoked by nerve injury. Moreover, it diminishes the level of p38 kinases and the level of two chemokines, CCL2 and CCL5, which are known to be strongly involved in the development of mechanical and thermal hypersensitivity. Our findings and available clinical data suggest that mirogabalin can be successfully used to treat patients suffering from neuropathic pain.

## Data Availability

The data presented in this study are available on request from the corresponding author.
